# Dr. Littlehales and Dr. Fowler, on the Influenza

**Published:** 1803-11-01

**Authors:** Thomas Beddoes


### Medical and Phyfical Journal.

VOL. X.] NOVEMBER 1, 1803. [NO. LVII.

*Printed for R. PHILLIPS, by W. Thorne, Red Lion Court, Fleet Street, London.*

### *To the Editors of the Medical and Phyfical Journal.*

GENTLEMEN,

I Send you an additional packet of Reports on the Influenza; among which I think you will find some as interesting as any that have ever appeared on the subject. I may yet receive a few more, and they shall be forwarded for next month, with my own observations.

In the last number, pp. 291 and 294, the proper name should be Giddes. The omission of the queries before each paragraph makes Mr. Tudor's letter read unconnected. The queries related to the facts, shewing the influenza to be contagious or otherwise, to its resemblance to catarrh, and its gradation from pneumonia to low fever.

I am, &c.

*October* 10, 1803. THOMAS BEDDOES.

90. Dr. MACKIE, Southampton, Sept. 16, 1803.

Southampton and its neighbourhood were uncommonly favoured; in February, March, and April, we had a very general catarrhal indisposition, yet by no means so universal that we should have given it the name of an influenza, if we had not heard so much of it from almost every other part of the Island. I believe it was sufficient, however, to fill the hands of our apothecaries. Few cases were so serious as to fall to the lot of the physician; those I saw, were marked with little or no peculiarity different from the common diseases of that season, and I neither met with, nor heard of, any that terminated fatally. I understood it was more severely felt in our neighbouring large towns of Portsmouth, Winchester, and Salisbury, from which you have probably obtained reports.

91. Dr. Littlehales, Winchester, September 14, 1803.

In answer to your inquiry I beg leave to inform you, that, as far as I am able to recollect, the influenza made its appearance in this city and neighbourhood about the first week in March. In a very few patients some serious peripneumonic symptoms occurred, but I have reason to think that, comparatively speaking, the disease was light in these parts, at least if I may be allowed to judge from a family who brought it with them from London, which suffered more severely than their neighbours. There was frequently so great a degree of direct debility that I was under the necessity of allowing wine diluted with decoction of bark, or camphorated mixture with a few grains of myrrh, and I did not perceive that it ever augmented either the cough or difficulty of breathing. Opiates were much less efficacious in this than in common coughs, and I sometimes thought the cicuta answered better. Blisters were always I think useful. The most remarkable circumstance was a continued want of appetite for a very considerable time after the disorder had entirely subsided. These are all the observations which occur to my memory, and I wish they were of more importance.

92. Dr. Fowler, Sarum, September 18, 1803.

This should have been with you earlier if I had been more successful in ascertaining dates. I saw no case of influenza before the 12th of March, nor any since the 8th of June. Mr. Wyche, who is a surgeon here, states his first case to have been seen on the 9th of March, and his last on the 11th of May. All the cases that I saw within the first two days of the disease, were instantly relieved by calomel and James's powder. I always applied blisters where there was pain in the chest; and where the inflammation of the larynx and trachea was such as to produce symptoms resembling croup, leeches were applied with good effect to the neck. I employed the lancet only in three cases. The symptoms differed from those of common catarrh in degree rather than in kind. The pain in the head was more severe. The sense of suffocation much greater during the first three or four days. In general, this symptom appeared to be produced rather by a spasmodic than an inflammatory affection of the larynx.

The first patients whom I saw with influenza were six interesting children in the same family. I then had the disease myself, but not so severely as to confine me. One large large family in the country, and who had little communication with others, escaped the disease till June. They thought they caught it from their music-master. Seven persons, who attended in succession a lady who had it severely, were attacked with it. Her daughters, who were kept away from her, escaped. Several instances of a similar kind occurred. Nothing that I witness authorises me to say *positively* that the disease was contagious; but I thought it right to separate as much as I could the sick from the well.

93. Mr. Earnest, Sheffield, September 18, 1803.

According to your request I send you the following account of the influenza, when it appeared in Sheffield, and adjacent places.

It commenced first of all in the neighbouring villages near and southward of the town of Sheffield, in the beginning of March; about the middle of the same month it appeared in Sheffield, and in April it became very general and prevalent. The symptoms in some cases ran alarmingly high, and was with great difficulty subdued. I have been informed that several cases ended fatally, but it was with such subjects as were predisposed to phthisis. I believe many practitioners have said that the influenza is a contagious disease, but several of the senior practitioners here say not; and since I received your letter I have particularly inquired into the truth of this assertion, and the result of my inquiries is as follows. It is a well known fact by some of our medical men, that when two or three of a family were ill in the influenza, others of the same family slept with them and never took the contagion.

94. Dr. Mossman, Bradford, September 19, 1803.

The first time I heard of the Influenza was of its prevalence in the city of York; it then approached Leeds; and about the 25th of April it made its appearance in Bradford; from whence it continued its progress to the westward. In Bradford and its vicinity it was the most prevalent about the middle of May, and disappeared about the end of June.

Its first approaches resembled those of a common continued fever; but its future progress was characterised by several peculiarities. The lassitude, chills, and succeeding heats, which mark the presence of fever, preceded the influenza; but in the influenza the pain in the head was

 not not so acute as in a common fever, though it was very distressing if the patient was put in motion; and very frequently accompanied by a confusion and staggering; pains resembling those of rheumatism were generally complained of, more especially in the larger joints. In every case there was an affection of the chest, accompanied by cough and dyspnœa, without any violent or fixed pain. The pulse was from 100 to 120; thirst considerable. In short, with a few peculiarities, it approached nearer to the features of peripneumonia notha, (as described by authors) than any other disease. The symptoms were very generally alike; they only differed in degree; and it was never fatal unless it attacked asthmatic or phthisical people, or those labouring under other diseases. All ages and constitutions were attacked by it; but females seemed to suffer the most severely. In the commencement of the disease, *emetics* were of the most essential service. *Local bleeding* and *blistering, nitrous* and *saline* medicines, with *opiates* at bed time, were the powers employed here, and they never failed to afford relief; *but the most effectual of all other remedies was the digitalis,* administered in the dose of 15 or 20 drops three or four times a day. *In the cases of children its operation was strikingly beneficial.* A low regimen was universally advised, with cool drinks, and a cool temperature. It was generally terminated by secretion from the membrane of the larynx and expectoration; but unless much care was employed, relapses were by no means unfrequent. The convalescents recovered very slowly; they continued long in a weak state; and it was remarkable that their intellectual powers were very often considerably impaired. When the inflammatory action was subdued, we advised nutritious regimen, and we employed *barks,* and the *vitriolic acid.* The disease was very certainly not contagious. During its epidemic influence no other disease appeared. Since the appearance of my pamphlet on Consumption, &c. I have had very numerous applications for the cure of that disease; and more especially since the prevalence of the influenza. Seven out of ten of my phthisical patients date their complaints from the attack of the epidemic in question; and I certainly think that there is a greater tendency to fatality in the cases originating in the influenza, than in those which have sprung from other causes. I find extreme difficulty in combating the symptoms; and the powers which I employ successfully in other cases, do not effect the same relief in phthisis arising from the influenza. This is curious; but I have no doubt of the fact.

No

No other disease, with the exception of pulmonary consumption, has appeared in this neighbourhood, which can be traced to the influenza.

I hastily submit to you these observations. You may make what use you please of them. They are thrown together without any proper arrangement; but you may depend on the facts.

95. A Correspondent, York, September 20, 1803.

On conversing with many medical gentlemen here, I am enabled to ascertain that the influenza was prevalent in York during the months of February, March, April, and May. Some catarrhal fevers were observed from about the middle of January, but were not then considered as a peculiar epidemic, although they very much resembled the disease afterwards called influenza. It was very general all February and March, and began to decline in April; there were however many similar complaints here and there through May and June, and perhaps still later.

The complaint was on the whole milder than in some other situations, and generally yielded to lenient methods of cure. Venesection was rarely necessary; and blisters were not often essential, but sometimes applied with good effect. The lower orders of the people must have managed it without much medical aid, for I am informed that there never were a great many patients in this disorder at the Dispensary; and it appears that the first applicants were so late as the 1st of April, when the vigor of the epidemic was somewhat abated in the town. Many suffered relapses, especially from exposure to cold; and one medical man had so many relapses that he did not get free of it for near six weeks. Happily for him he is in the vigor of life, and of a good constitution. It has not been considered as a contagious disease by the greater number of the practitioners in this city. Many instances have occurred where a whole family have had it in a most rapid manner; many where only one or two in a family, and others where the husband has escaped although cohabiting with his wife, who had the influenza; and vice versa. Some numerous families had it not at all, although no less intercourse than usual existed. Infants, in general, suffered more than adults; in some cases repeated bleedings with leeches and emetics were required. Old people, and especially those who had laboured under pneumonic affections, suffered much; and a very few died. Vertigo, to an excessive degree, occurred in a small number of cases, in which the other symptoms were not considerable. A more minute account of symptoms, or detail of the method of cure, would be superfluous, as they agree pretty much with what has been pursued very generally. I shall only add, that two practitioners here thought the digitalis very useful in the commencement of the complaint.

The above seems all that is necessary as to what regards York, which I should have been glad to have sent you sooner; but you must know that it is no easy matter to collect the report of ten or twelve medical men, who have to look back into day-books, &c. In a few days I shall be able to send you a farther account of its appearance, &c. &c. in a circuit round York, whose radius cannot be less than thirty miles.

The same Correspondent has taken the trouble to explore the district surrounding York and has given the geographical positions so accurately, that if we had many similar examples, nothing further could be desired as to the progress of the complaint. The following nine numbers comprize his collection. Nos. 96—104.

At Kirby-moor Side, about thirty miles to the N. N. E. of York, situate at the edge of an extensive tract of barren moors, which separate it from the sea from north to east, Mr. H. observed as follows.

It is with extreme regret that I have at so distant a period to acknowledge the receipt of your last in September. Having had numerous and distant professional calls, I have been thus long prevented. I am half persuaded that any observations that may fall from me will be too late for the purposes designed, if not too trivial for further communication; yet, that I may not withold my mite in settling the present controversy, or be thought indifferent to a request which you have honoured me with, I now hasten to observe,

That the influenza made its appearance here in April, and continued in May and June; that several passed through the disease in so mild a manner as not to require medical attendance, yet a few solitary cases proved violent, and put on alarming appearances. Few or none however proved fatal, except in the case of a maniac who expired in a sudden and unexpected manner. About this period the typhus mitior prevailed in this vicinity, together with several irregular cases of pneumonia, in which two asthmatic patients of a middle age were snapped off suddenly; and I am sorry to add, that a more than usual proportion of of cases resembling phthisis, have, since the prevalence of the general epidemic, been observed by me, which I can but with others (in predisposed habits) look on as a sequela thereof. The influenza bore a very considerable resemblance to a catarrhal affection which prevailed here in October and November preceding, but without the pre-exciting cause, viz. exposure to cold, watching, or fatigue. In its attack it was very different; instead of introducing itself slowly and insidiously, its commencement was immediate and sudden; pain of the forehead and temples, the eyes more or less affected, slight dyspnœa, accelerated pulse, pyrexia; these were soon followed with the variable characterizing symptoms of low fever, accompanied with such an extraordinary prostration and general debility of the whole system, as served to correct the mistaken ideas of those who had previously looked up to bleeding as a necessary measure. A petulant cough, which was seldom attended with free expectoration, was more or less distressing, unaccompanied with that coryza we experience from acquired cold, a dejection of spirits, and sometimes erratic muscular pains in the limbs and different articulations. These, together with a confusion of mental intellect, and a very slow return of strength, were in various combinations generally present, and which in my idea was what constituted our late general epidemic. Emetics at the onset; diluents; the free use of aq. ammoniæ acetat. sometimes combined with mistur. camphor. epispastics; mild aperients; tinct. opii. camphor. and sp. vitriol. d. were employed with general advantage.

I come now to give my opinion as to its contagious powers; and am sorry to observe, that from the division that prevails in the sentiments of the Faculty, the common adage so frequently applicable to another profession may attach to ours, and that the "*in*-glorious uncertainty of *Physic*," may be as proverbial as that of *Law*. Allowing, as I am ready, that several in one family have suffered nearly together in the complaint, whilst on the other hand one or two only out of a great number have been attacked, this confirms my idea that we are still to look for one common exciting cause, viz. atmospheric influence; but before this can operate there must be a peculiar idiosyncrasy or aptitude in the constitution to receive this morbid impression; and wherever these two causes exist I would venture to suppose the result will be uniform, and the general endemic, alias influenza, the consequence. I cannot suppose the complaint is ever the offspring of contagion arising from human infection or fomites; and indeed to countenance this opinion it is necessary to allow the duration of the complaint to exist for a longer period than it has been observed in the system, before such an assimilation or congeries of miasmata could pravail, so as to render it eminently infectious, at least if we are to infer from analogy with other diseases that have proved communicable from one individual to another. I am well aware that these arguments have been embraced for espousing both sides of the question, and shall anxiously look for Dr. Beddoes's suming up a commentary from the great mass of testimony that has been already before him and the public.

You are perfectly at liberty to make what use you please of this; I regret that it is not as I could wish, more *succinct*; but if your packet be not already dispatched to Dr. Beddoes, and you should deem it of any consequence, perhaps you might abstract such parts from it as you may deem worth the trouble. I cannot close this without expressing a wish that Dr. Beddoes may favour the world with a nosological arrangement, or classification, of the present disease, as a lasting memorial of his attention to the epidemic, which at various and distant periods has so suddenly diffused itself across this island.

At Tadcaster, nine miles south-west of York, where the vale of York begins to terminate in a variety of rising grounds, Mr. Shann informs me the first patient he saw was on the 15th of March; "if I had seen any before that time they had been considered as common catarrh." From the 20th to the 28th I saw many families, in each of which, six, eight, or ten were seized with the complaint in twenty-four hours; "at a later date I think the epidemic became less rapid in its progress, but continued to spread gradually till about the middle of April, at which period few cases of the disease remained. In more than two or three instances, in large families, I observed most of those escape who were not attacked with the complaint within three days of its first appearance in that family, although they waited upon those ill of the disorder." During the progress of the disease, Mr. S. observed, especially in two instances, where it had not yet been in the family, that in less than thirty-six hours after the arrival of an invalid, (in this complaint) from a short distance, two persons in each family were attacked by it.

The prevalent symptoms were pyrexia, acute pain accompanied with a sensation of tightness across the forehead; oppression of the heart with a troublesome cough; and and in many instances, especially in those who were in the habit of free living, great depression of spirits.

Antimonial and saline medicines, with occasional purgatives, appeared the most useful. Tonics and cordials in very few cases were indicated; and when tried, commonly seemed to aggravate the most troublesome symptoms.

At Selby, fourteen miles nearly south of York, in a very flat country, Mr. Fothergill first observed the complaint about the 12th of February. The symptoms first appeared truly inflammatory, attended with violent pain in the head, and a disposition to vomit. After an emetic, antimonials with saline mixture gradually reduced these symptoms in five or six days. General debility ensued with a slight appearance of putrefaction, attended with great irritability. Mr. F. found the cinchona very useful. The complaint lasted twelve or fourteen days, and in some instances longer. Seldom or ever fatal. In many instances the whole family were more or less afflicted. *Some had an eruption upon the skin, attended with sore throat.*

At Howden, twenty miles south east of York, in a very low country, Mr. Whiteley saw the first patient the 1st of April; the last, the 14th of June. The complaint appeared inflammatory the first two or three days; then followed excessive depression of spirits and prostration of strength. In some cases venesection seemed indicated; but although it relieved the symptoms, it very much increased the depression and prostration of strength; these last symptoms were cured by the cinchona. The complaint lasted from two or three days to a fortnight. Mr. W. saw one hundred and fifty cases, of which two died, both old people, of infirm constitutions. The severest symptoms were pain in the head and back; in some, an inclination to vomit and pain in the stomach, were always relieved by an emetic. Mr. W. had heard of the complaint both at Hull and at York before he saw it. Its progress was extremely rapid for the first fortnight or three weeks. Those most exposed were most subject to the attack; more adults than children. It was always attributed to cold by the patients themselves. Measles preceded this epidemic. Mr. W. did not observe that pneumonia, the most prevalent complaint in the spring months, was more frequent or more unmanageable.

At Hutton Bushell, about six miles from Scarborough, Mr. Smart saw the first patient the 3d of April, and the last the 6th of May. It again made its appearance the 13th of August, on which day Mr. S. was called to six patients; three in one house. It ceased about the 27th of the same month.

month. "The disease began very suddenly, with cold tremors, sensation of cold water trickling down the limbs; then followed heat and sweat, and dejection of spirits. Pain in the head, especially in the orbits of the eyes. The albuginea appeared red, but its vessels were not turgid. Pain in the breast with cough, which went off without expectoration. In numbers the pain in the head and breast alternated. When great dejection of spirits prevailed, the sick complained of a dull heavy pain, like a weight fixed upon the back part of the head. Pains like rheumatism or erratic gout were felt in the extremities, but never continued more than two or three minutes at a time in one part, or in the same limb. While these pains occupied the limbs, the head and breast were relieved. Pulse little disturbed; tongue covered by a yellowish crust; thirst never distressing. Mr. S. never bled. Nor did he lose a patient. He administered an emetic in all cases, and where necessary an opening medicine; and conducted the cure with antimonials, &c. His best cordial was a limited allowance of Port and water. The cinchona in no instance seemed to do good. Mr. S. never had recourse to a blister, so much recommended; but does not know any case in which a complaint of the lungs was left behind. During the prevalence of the influenza in the spring, we had mild weather succeeded by easterly winds, which are very severe on this coast. The epidemic abated as soon as these cold winds began to blow. Sore throats then were frequent, by many called influenza, but wanting the characteristic symptoms of that disease. Mr. S. saw many sore throats whilst influenza prevailed; but never in cases of true influenza did he see sore throat. During the time this epidemic prevailed in August the heat was intense and the country burnt up. Scarlatina anginosa and pertussis succeeded the first appearance of the influenza. The former continued to prevail about three weeks; of the latter there are still some instances, (Sept. 18). Two of them are in the same house; a child and an adult. "In this house the influenza prevailed in the spring; but neither of my present patients had it. Nor do I recollect that any patient has had the influenza, who had been previously attacked by scarlatina or hooping cough: or vice versa." Mr. S. did not at the time make inquiry whether the influenza patients had previously had either of the other diseases. It is worthy of remark, that in villages near the sea, the scarlatina prevailed before the influenza began, seemed suspended suspended during the epidemic, and first appeared again in those very villages.

At Easingwold, thirteen miles north of York, approaching the high hills of Hambleton, Mr. George Cock observed the influenza made its appearance the latter end of March; it became gradually more general till the middle of April, and by degrees declined. The first symptoms were slight chilliness and shivering, soon followed by violent rigors and alternate heat; the pain in the head, especially in the forehead, now became extremely distressing; shortness of breath, tightness and pain in the chest, with harrassing cough, hardly admitting relief; scanty expectoration; coryza; the discharge from the nostrils excoriating the lips; an uncommon irritable state of the stomach also supervened. Except in a few delicate females the pulse was hard and corded; in most cases strong and bounding, from 100 to 120 beats in a minute; the tongue white and parched, with cracks; occasionally smeared with dirty looking mucus; thirst very great, urine clear and high coloured, with every mark of excessive excitement. The predisposed to pulmonic disease had hard struggles. "In almost all cases, I found it necessary to use the lancet, and often very freely; in one instance, twice in twelve hours, fourteen ounces each time. The blood always exhibited the buffy coat; and as soon as it began to flow, the pain both in the head and breast was generally relieved, and the patients said they felt *lighter*. In hardly any case did I find it produce the debility medical men have so much remarked and dreaded in this disorder. In weak females I found six or eight leeches frequently applied to the temples and breast alternatively give great ease, where general bleeding was inadmissible." A grain of tartarized antimony and twelve of ipecacuanha operated well, and brought up a large quantity of bilious matter, which relieved the sickness. Blisters applied to the breast and between the shoulders were of great service. Small doses of pulvis antimonialis with saline mixture, opiates at night, and attention to the alvine discharge, generallly terminated the cure. "After the urine began to deposit a sediment, the bark and other tonics were of the greatest service. I only saw one fatal case, which was an old man, who had been a sufferer by thoracic inflammation many times before." Mr. C. had no reason to think the disease contagious.

At Thirsk, ten miles beyond Easingwold, to the westward of the Hambleton hills, Mr. Wasse informs me, the complaint complaint was more mild than in other situations. He had no patients under his care whose illness gave him much anxiety. The disease made its first appearance in his practice on the 23d of March; "it was confined to six or eight cases until the 10th or 11th of April, when it suddenly became very prevalent; more so I think in the villages and farm-houses than in Thirsk." It appeared more general about the 26th of April; and before the 15th of May had pretty generally declined. "During this time I saw a great number of patients, most of whom required no medical aid at all. I only used the lancet in one case, a strong middle aged man, who sunk more after the operation than I expected. In a few cases I applied blisters where the lungs seemed more than usually affected, but found the complaint very readily give way to small doses of tart. antimony, with pulv. ipecacuanha, &c. The symptoms sometimes began to abate in a few hours; in a day or two were generally much lessened, and in six or eight were entirely gone, leaving the patients uncommonly much weaker than I expected. The predominant symptoms were an incessant and violent cough, with slight pains in the chest, rigors, languor, and what I conceived characteristics of the disease, a peculiar white tongue, and a violent distressing pain in the head; pulse seldom so much affected as might have been expected from the urgency of the other symptoms; extreme thirst was very rare." The circumstance of this disease becoming general in the whole district as it were at once, and whole families seized at the same time, Mr. W. thinks, does not support the idea of its being contagious.

At Masham, about thirty miles N. W. of York, and twelve or fourteen west of Thirsk, in a very high and mountainous country, Mr. Baines observes, that the influenza made its appearance about the middle of February. It was not however distinguished by that name till later, nor did Mr. B. begin to compare it with the influenza which prevailed in April and May, 1802.

"The general symptoms were pain across the forehead with vertigo; shivering or chilliness, generally in the afternoon; obstruction of the nostrils, with inflammation of the pituitary membrane; dryness and soreness of the fauces, and other catarrhal affections; sickness, and an inclination to vomit, for two or three days; pulse about 90, seldom quicker; tongue white, but moist; little thirst; bowels for the most part costive; urine higher coloured than usual, and generally letting fall a copious sediment; on on the third day a pretty free discharge taking place from the mucus membrane of a greenish colour, without smell; severe cough. Those who coughed severely, complained of great pain across the thighs, in the arms about the insertion of the deltoid, and in the sides. Unless the disease made an attack upon the lungs, the patient was never confined to the house, nor were his symptoms exasperated by his going abroad. Those whose illness rendered confinement necessary, had all the appearance of pneumonic affections. Yet, although patients of this description expectorated much, I never perceived the least tinge of blood. The discharge had rather a purulent appearance. In all cases whatever, the complaint continued, after the first three days, much the same, till about the fourteenth day, when the patient gradually recovered, but so slowly that a month generally elapsed before he was quite well. Very few died, and those who did, died by suffocation. The influenza raged most in April and May. After the month of June few persons applied for medical assistance; an idea about that time beginning to prevail that the disorder must have its course, and that waiting patiently was all that was necessary."

Mr. B. observes, " That when the influenza made its way into a family or village, it generally spread through the whole of it. On the other hand, I never was called to attend a patient living in a high situation; nor have I ever heard of its being known in the villages situate on the sides of hills, of which there are many in this neighbourhood. Mr. B's mode of treatment coincides pretty much with what has been already mentioned. It does not appear that he used the lancet.

Mr. Smart, of Hutton Bushell observes, that in one case a person employed to shave another, beginning in the influenza, although this circumstance was unknown to him, went home and complained of the disagreeable smell of his customer's breath, and in less than three hours was attacked with the disease; had it very violently; but his wife, who slept with him the whole time of his illness, escaped. Mr. S. met with several similar instances. In almost every instance, irregularity or excess, especially in drinking, caused a relapse. " In one person who was convalescent I ordered red port and water without specifying the quantity, and when I visited the next day, found he had taken the moderate allowance of *two bottles*, which brought on a relapse that had nearly proved fatal."

*Comme j'ai encore un peu de blanc,* observes the anonymous collector; I will add, that I have executed your request in the most ample manner I conveniently could. I regret the delay; it is not, however, chargeable to me—you will therefore acquit me of the imputation. The details of this disease are curious in several points of view. It is undoubtedly the same complaint; and whatever latitude we allow to our fallible means of observation, it has been singularly modified by extraneous circumstances. How different the complaint at Easingwold and at Thirsk, only ten miles distant, and in many particulars, especially in situation, &c. not very dissimilar. If, however, we inquire into the seat of the disease anatomically, we shall find it, I believe, universally the same, and only varying in degree. The *mucous* and *fibrous* membranes seem chiefly to have been affected by the influenza. The former, covering the inside of the mouth, nostrils, anterior parts of the eyes and eye-lids, fauces, trachea, and all its pulmonary ramifications, œsophagus, and stomach, &c. and the excretory ducts of the numerous glands which open upon its extensive surface, were universally affected. Of its effects upon that part of this membrane which lines the intestines we have little evidence. It has apparently had some effect upon the biliary and probably upon the pancreatic ducts. Large portions of the *fibrous* membranes, especially the dura mater, linings of the orbits, the ligaments, fascia and strong folds, which separate the muscles, and perhaps the periosteum itself, may, without improbability, be considered the seat of some of those erratic or more durable pains so frequently characteristic of this disorder. In cases of greater severity it is probable the affection has been extended, especially in the predisposed, to the *serous* membranes, particularly those forming the lining of the thorax, and the covering of the lungs. With respect to the question of contagion, there are too many points unsettled to admit of my touching upon it at the close of this long paper. All the diseases commonly acknowledged to be contagious, seem to require something extraneous and in addition to the poison itself, in order to produce that spread we call an epidemic. The history of the plague and American fever, by which we learn that they neither of them rage at all seasons, seems to support this inference. The former ceases to extend its ravages at a particular time, although little or no precaution is taken by the inhabitants of Egypt to arrest its destructive progress. And to whatever degree the the people of America may be supposed to carry their efforts for that purpose, they do not secure their large cities from feeling, at the return of the season, the inefficacy of them. Facts of a similar tendency occur not unfrequently in measles, scarlatina, and hooping cough, and I believe also in small-pox; I can therefore readily suppose the influenza infectious; so may be common catarrh, and yet requiring something more to become epidemic.

105. Mr. Fieldhouse, Stafford, Sept. 22, 1803.

Mr. Ward and myself are fully convinced of its being highly infectious, and ground our opinions upon the following circumstances among many others which occurred.

Mr. Ward's family living in the country, and detached from any house, continued free from the complaint until a late period, when Mrs. Ward paid a visit to some ladies who were ill in Brewood; she was attacked in three days after being in the family, and returned home. About the same distance of time, a little niece that they kept was seized; the child hanging about Mr. Ward, he was taken ill, with a lady and the rest of the family. A schoolmaster, in Brewood, (a small town) kept his scholars and family unconnected with the rest of the inhabitants for a considerable time, and till the complaint had nearly disappeared; during this period they continued perfectly free, but upon taking off that restriction and mixing with the people, they were all soon attacked. A ladies school that I attended kept perfectly free by the constant use of the nitr. & ol. vitriol, notwithstanding the complaint raged at the next door; being a school lately begun, possibly they took more pains in using the fumigation; certain it is, however, that family never had it.

How far these cases may tend to clear up the matter I know not, but this you may depend upon, that they are facts.

106. Mr. Wilkinson, Sunderland, Sept. 28, 1803.

The first appearance of the epidemic catarrh, occurred on or about the 6th of February; prior to this period, the hooping-cough had greatly prevailed among children, and in some instances proved fatal to those of younger years. Some cases of cough, accompanied with slight catarrhal symptoms, took place in persons advanced in years, particularly those who were predisposed to asthmatic affections, or what is usually termed the *tussis senilis.* These last last were relieved by the application of blisters to the chest, mild pectoral anodynes, and the external use of flannel.

Some were attacked with colicky complaints of the bowels, accompanied with diarrhœa and gripes, which were relieved by sudorific anodynes, the occasional use of *ol. ricini,* and the same external covering.

From the 15th of January to the period in which the epidemic catarrh took place, several cases of *cynanche tonsillaris* fell under my observation; and in two or three instances, where the tonsils were not much tumefied, were accompanied with slight cough, pains in the chest, and some degree of *pneumonic* inflammation.

As the month of February advanced the sore throat went off, and the disease continued to increase gradually, I mean the *influenza.* It attacked a considerable number of people at once, in March, and went on progressively to the latter end of April, when it gradually diminished as the weather became warm. In May, however, some solitary cases took place, which assumed the typhoid form; even in June several instances occurred; and towards the latter end of July, myself and another person were affected, which confined me two days in bed, and some days in the house. The most urgent symptoms were, great depression of strength; pulse from 90 and sometimes to 120; pains in the limbs, particularly the loins and head, the latter was always the most distressing; in some, considerable difficulty of breathing took place, with incessant cough, and *pneumonic* inflammation. In others the appearances or symptoms varied much, and were very mild, so as neither to confine them in bed nor within doors. In the parish work-house which I attended, where there were about 280 paupers, consisting of every age and sex, five were at once attacked on the 7th of March; the day following seven more became ill, and a day or two after thirteen others were affected; afterwards it continued to go on, so that in the whole, not more than thirty-seven, or at the most forty patients out of this great number catched it.

On the 8th I saw seven patients in the Seamens' Alm's-houses, which are in an open airy situation, forming a small square, containing in number about thirty persons, the rooms rather small, but not inhabited by more than two persons. These had been suddenly attacked a day or two before, at one and the same time; five were men, and two women; their ages varied from sixty to seventy years; the former laboured under violent *pneumonic* inflammation with symptoms of pyrexia, &c. and bore V. S. remarkably well, by by which, and the use of blisters to the chest, antimonials, and mild anodynes h. s. they very soon recovered. Four more fell ill of the disease; the proportion was seven men to four women. The women and two of the men were but mildly affected. No others out of the whole number caught the complaint, although the women and their husbands slept together.

Generally speaking, it evidently appeared that the symptoms of direct debility were more predominant in persons under forty or fifty years of age; than those younger, in them the inflammatory symptoms were more strongly marked, particularly in men. In the work-house none of the patients were bled; the disease in the men having taken on the appearance of typhus, and they recovered more slowly, while the women were more mildly affected, except in two or three instances, where the disease terminated in *phthisis*. In these, however, a predisposition to cough seemed to occur prior to the attack of the disease. Children in general, especially those under eight or ten years of age, were but little affected with this complaint; and what is not a little remarkable, this was the case with those in the work-house, in which there were a great many: several had laboured under the hooping cough; and as the warm weather advanced in June, July, and August, many were seized with measles, and some with scarlatina anginosa, from which they recovered with little or no medicine.

In no one instance under my care do I recollect that this disease, simply considered, proved fatal, except in one case, where it attacked a woman in her *accouchement*. Pregnant women appeared to suffer more than others; in them the inflammatory symptoms, such as cough, dispnœa, attended with pneumonic affection, were more strongly marked; hence it appeared that moderate v. s. and that well-timed, became in them a *sine qua non*, and in all other instances where such evacuation was indicated, it always proved beneficial. It may not be here improper to observe, that in almost every case where blood was drawn, that it assumed the cup or concave appearance of the coagulating lymph on its surface. In two or three cases, where the inflammatory symptoms nearly approached to suffocation, and which occurred in patients that were previously subject to inflammatory affections of the thorax, bleeding was repeated two or three times, and to this I attributed their recovery. In these I had hesitated to use the lancet at the commencement of the disease, from an idea of depressing the system, a fear to which many practitioners were perhaps subject, whereby not a few have been lost, that otherwise might have survived. It did not appear that any age or class of persons, if we except young children, were less exempt than others, nor were those within doors less liable than those exposed to the open air; and the proportion of males to females affected, so far as I can discern, were nearly on a par. Mariners were not uncommonly attacked at sea; some of these were affected at that period with syphilitic complaints, and were under an alterative course of medicine; on shore, the same effects ensued with perons under smilar circumstances. In some instances where the disease was nearly cured, as in gonorrhœa and inguinal tumours nearly dispersed, these seemed to reappear, or were otherwise aggravated. In general it happened, that whatever might be the indispositions to which certain persons were habitually affected, these were aggravated even where no appearance of influenza existed; but when it took place, and continued for some days, it seemed to participate of typhus.

Some females, particularly from forty to sixty years of age, where menstruation had ceased, were violently attacked with acute rheumatism, such as tumefaction of the wrists, knees, ancles, &c. accompanied with symptoms of pyrexia; in these the catarrhal affections were more mild and generally unattended with *pneumonic* inflammation: they gave way to the lancet. Antimonials combined with calomel, sudorific anodynes at bed time, and the decoct. cort. salicis in the remittent state of the disease being given, greatly facilitated their recovery. In two cases I had previously exhibited the *decoct. cinchonæ* with a view to determine its comparative powers with the former; but although it was taken freely for more than two days, yet no amendment took place, while in those who took the decoct. salicis, under similar circumstances, prior to using it in these two, the febrile paroxyms and recurrence of the pain were speedily prevented.

Of the efficacy of *this* decoction in the speedy recovery of those patients already named, I am the more convinced, as at that time I well knew some female patients affected in like manner, who were treated nearly in a similar way, and took the cinchona without benefit. One of these, after being ill for near a month under the care of another gentleman, consulted me. She was treated in the way already noticed, venesection being premised on account of the symptoms of pyrexia still remaining; these being removed, with the assistance of the salix decoction, she was perfectly ly free from her complaint in eight days, but continued it for two days more. Before I became acquainted with the *salix*, I had repeatedly tried the *cinchona* in similar situations, but it fell far short of it. Relapses were not very common, or rather few had the disease twice, except where they were incautious in exposing themselves too soon in the open air; I do not recollect more than half a dozen, of 120 whom I attended, in whom the disease re-appeared; but in these it was usually more violent.

The treatment I adopted was, venesection where indicated, but this I used only in urgent cases, combined with inflammatory symptoms, or in peripneumonia, *pulv. antim. gr. ij. ad iij. calomel, gr. ad ij.* 6 *quaq*; *hora.* Vesicatories, and these often repeated *sterno vel scrobiculo cordis,* gentle anodyne sudorifics h. s. to abate the violence of the cough, and the salix decoction in the intermittent or convalescent state; the use of wine according to circumstances, in sago, tapioca, or Indian arrow root; and as the strength and appetite returned, light animal food, &c. I never had recourse to emetics, as the alteratives in the form of a bolus already named, where nausea or sickness prevailed, generally acted in this way, and where constipation existed, usually produced starts, exclusive of their general effects on the system by exciting a copious perspiration. In some cases this remedy, as is well known, has acted in these three ways at once, with infinite advantage to the patient, and that with more certainty than antimonials singly.

This epidemic, to the best of my recollection, as I was then a young practitioner, bore some resemblance to that in 1782, which commenced in the latter end of May, and continued till near the beginning of July. It was I think more generally diffused, and more violent in its effects; it appeared to be less combined with *peripneumonic* inflammation, and not so much to require the lancet. Several died, but many fell victims to phthisis pulmonalis, while great numbers, as in the present, were but slightly affected.

A similar disease took place in 1788, but this participated more of the mild catarrh, and it was not so general in its progress, nor alarming in its effects. An epidemic sore throat prevailed nearly at the same time, but this was not very dangerous, neither did the catarrh require bleeding, being less inflammatory.

To determine precisely whether the epidemic disease of which we are now treating be contagious, is what I shall not attempt, although in that which occurred in 1782, I entertained but little doubt that it was. But this opinion

 was was formed more from a general belief of its being so, and the manner in which its progress was traced, than from any idea I at that time had formed of my own. Entering into a discussion pro or con on the subject, is what I am totally averse to, as this must be referred to more decisive facts or positive proofs, than has been to *me* hitherto advanced. The following extract of a letter just received from a friend, an eminent physician in the county of Norfolk, I beg leave to insert, in which although nothing strictly positive is inferred; yet the difficulties with which it appears to be involved, are so candidly set forth, that it would seem not so easy a task to decide on this question, as either party have so positively asserted.

"It is very hard to produce facts for or against the influenza being contagious. I have known so many intances where a patient has died of the *typhus gravior*, without any one of the family suffering the slightest symptom of fever, that no argument can be drawn from the circumstance of only one person in the family having had the influenza. But I think that of whole villages on the Norfolk and Suffolk sides of this country being attacked in one night's time with the disease in question, depended upon what the old writers call an *occult* quality in the air, and not on contagion. If persons be exposed to the same air, diet, and fatigues, they are subject to the same diseases, independant of infection communicated from one to another. Soldiers and officers, whether they use public or private privies, become in the same proportion affected with dysenteries, when they are equally exposed to the same camp fatigues and diet. Very few medical men have had so good an opportunity as myself of proving this fact.

"The luxury of an Indian army is such, that an officer has generally a small tent to himself for a privy; yet a similar proportion of *these officers* were on my sick report labouring under dysenteries, as of those privates who had the public privies to go to. But it is easy to argue against this conclusion, though the fact be admitted, Nothing is easier than to raise doubts against facts which can be produced for or against contagion. Still I am inclined to think that the prevailing state of the air of a country has more to do with most epidemics, than the infection that is imparted from the sick to the surrounding air of a room or camp.

107. Dr.

107. Dr. Marshall, Lynn, Norfolk, May 15, 1803.

The influenza appeared here between two and three months ago; but whether it is to be ascribed to the state of the air, or to contagion, doubts have arisen.

From *tolerably good authority*, it was said to have appeared in this neighbourhood, in some almost insulated situations, much about the same time that it was first remarked in towns. At this period, I observed instances where it was introduced, apparently, by contagion into families, through which it seemed to be gradually propagated. In some instances, it was ushered into families, but made little or no progress.

Upon the whole, considering the almost innumerable means of intercourse now introduced into most parts of this island, I am inclined to think that it was the offspring of contagion; although, from the invisibility of the poison, it was extremely difficult in some cases satisfactorily to trace it. This is most certain, that neither the thermometer nor the barometer indicated any thing unusual in the state of the air.

The disease certainly ended sooner in towns than in the country; and so far as my own experience extended, it began sooner there also. This, as you justly observe, "*looks more like infection.*"

The disease, I have understood, first appeared in London, and it did not reach Edinburgh till two or three weeks after. This marks progress, and certainly favours the opinion of contagion.

The flying soil of Norfolk no longer exists. What was once the reproach of the county, is now its pride: By means of sheep and turnips, together with a spirited system of husbandry, it is completely arrested, and converted into fertile land.

P. S. Since I wrote the preceding, I have been informed from good authority, that in some of the distant hamlets, the influenza is not yet entirely extinct.

108. Dr. Duncan, senior, Professor of Medicine, Edinburgh, Sept. 25, 1803.

I think I can answer your question, respecting the date of the appearance of the late influenza at Edinburgh, with tolerable accuracy, as I have already had occasion to make diligent inquiry on that subject. It must however be allowed that influenza is very apt to be confounded with

 common common catarrh from cold, a very frequent disease in this country at every season, but particularly during the winter. From this circumstance, even the most discerning practitioners may be deceived with respect to the commencement of influenza.

The first well-authenticated case of influenza in Edinburgh during the year 1803, of which I have been able to obtain any accurate information, was that of Mr. M'Donald, of Powder Hall, who was attended during his complaint by my worthy friend Mr. John Walker, surgeon.

Mr. M'Donald, with his wife, his son, and his daughter, were in London in the beginning of 1803. They left London on the third of February, at which time the influenza was very prevalent there; but they did not know of their having been in any house where there were individuals subjected to that disease. When they set out on their journey they were all in perfect health.

They arrived at Berwick-upon-Tweed on the 8th of February, and were there in a house where there were several persons subjected to influenza.. They arrived at Powder-Hall, situated within a mile of Edinburgh, on the 9th of February. The next day, Mr. M'Donald himself was attacked with severe febrile symptoms, attended with uncommon prostration of strength, and all the other appearances which most frequently occur in influenza. Soon after Mr. M'Donald was attacked with this disease, almost every other person in his family, amounting to near a dozen, were attacked in succession. But its progress, as far as I could learn, was not immediately afterwards very rapid in the city of Edinburgh, and I did not myself attend any case where the disease was distinctly marked till the 23d of February, when I was called to a gentleman dangerously ill of the disease, several of whose family had before been affected with it in a much slighter manner.

By the end of February, the influenza was very common in Edinburgh, but as far as I could judge, both from my own observation and that of others, a greater number of patients were subjected to it about the end of March, than at any other time. It did not make its appearance in my own family till the 21st of March. The first of my family affected with it was my youngest son, a boy about nine years old, at the High School. At that time, many of his companions laboured under the disease. Six others of my family were afterwards affected with influenza in succession; succession; but neither Mrs. Duncan nor I were subjected to it, and two of my servants also escaped the disease.

When the influenza prevailed in Edinburgh during the summer of 1782, I had a severe attack of it; but Mrs. Duncan escaped that epidemic as well as the present, although all the rest of my family were subjected to it.

I observe by the Medical Journal of London, that several of your correspondents are inclined to think that influenza is not a contagious disease. I am decidedly of a different opinion. The contagion of influenza is not indeed conveyed on the point of a lancet, to be intentionally communicated like small-pox: But from all that I have been able to learn of the history of this disease, as recorded by eminent writers for many centuries past; from all that I have seen of it during former epidemics; from its progress during the present epidemic with very different states of the atmosphere, when passed from Paris to London, from London to Edinburgh, &c.; from its progress in Edinburgh after it appeared in this city; and finally, from its progress in my own family after its introduction into my house, I have no more doubt of the contagious nature of the influenza, than I have of the contagious nature of measles, chin cough, or typhus fever.

By the last volume of the Annals of Medicine, published at this place about the beginning of May, you will observe that very exaggerated reports respecting the frequency and fatality of the influenza, were propagated at Edinburgh. The accounts of the fatality of the disease in particular were very highly exaggerated. During the late epidemic indeed I attended several patients who were dangerously ill of the influenza; but I did not attend one to whom the disease proved fatal; and, in a great majority of cases, nothing was necessary but prudent attention to regimen. Indeed I am persuaded, that in the treatment of the late influenza, practitioners more frequently erred from doing too much than from doing too little.

I have now certain evidence, that the deaths from influenza were by no means numerous at Edinburgh. This is demonstrated by the register of burials in the Gray-Friars Church-yard, which you know is the principal burying place in this city. During the months of March and April, the numbers of burials from all diseases were each week as follow:

| | | | | | | | | | | |
|---|---|---|---|---|---|---|---|---|---|---|
| From March | 1 | to | March | 8 | — | — | — | 19 |
| From ditto | 8 | to | ditto | 15 | — | — | — | 25 |
| From ditto | 15 | to | ditto | 22 | — | — | — | 12 |
| From ditto | 22 | to | ditto | 29 | — | — | — | 15 |
| From ditto | 29 | to | April | 5 | — | — | — | 28 |
| From April | 5 | to | ditto | 12 | — | — | — | 29 |
| From ditto | 12 | to | ditto | 19 | — | — | — | 24 |
| From ditto | 19 | to | ditto | 26 | — | — | — | 20 |

Total number of burials for the eight weeks during which the influenza was most prevalent at Edinburgh. } 172

Although therefore it was positively asserted by some, and believed by many, that no less than one hundred persons, dying of influenza at Edinburgh, had been buried in one day, I am convinced that during the prevalence of this epidemic at Edinburgh, for the space of three months, the whole deaths from influenza did not amount to one hundred, when taken altogether.

The influenza however had probably some influence on the bills of mortality at Edinburgh; at least, during the first quarter of the year 1803, more burials took place in the Gray-Friars Church-yard, than during the first quarter of the preceding year. In the months of January, February, and March of the year 1802, the number of burials was 158; but during the same months of the year 1803, the burials amounted to 238. Thus there was a difference of no less than eighty, which might, I dare say, be principally attributed to the influenza.

109. Dr. Speer, Trim, Ireland, July 24, 1803.

The influenza made its appearance in this county the middle of February last, and disappeared the beginning of June. It first originated in towns, and afterwards spread gradually over the entire country, confined to no class of people. The inhabitants of low and marshy situations, and adults, suffered much more severely than those residing in airy or more healthful situations; the very heavy rains and moisture of the atmosphere which then prevailed, made both a variation in the symptoms and frequency of the disease.

People of debilitated or relaxed habits suffered much more severely, and were much more subject to relapses than those of a solid fibre. In fine, the symptoms of the late epidemic, epidemic, so far as I am capable of observation, were Proteus like, consequently the mode of treatment must have varied. The incipient symptoms in its first stage were excessively violent, and ushered in with universal debility and lassitude, *vertigo, nausea,* violent *ophthalmia, angina,* sometimes *cynanche maligna, excessive pain in the loins, loss of rest* and *appetite.* But in general did not prove fatal to any who were properly attended to, except adults and valetudinarians who previously laboured under pulmonary affections; on the other hand, convalescents suffered by extreme debility for a considerable length of time, which daily, among the lower class, terminates in dropsy.

110. Mr. Rewell, Cheltenham, October 8, 1803.

The influenza made its first appearance here towards the end of February. It was very general, but except in a very few instances, was extremely slight and hardly to be distinguished from the common catarrh.

In the cases which were more severe, there was a tendency to debility after the first three or four days with very considerable expectoration. In this stage of the complaint, I found it necessary to have recourse to tonics. The severe cases, however, were very few. I do not recollect seeing or hearing of any instance of its proving fatal. Very few cases occurred after the first week in April.

111. Mr. Thoresby, Holywell, North Wales, Sept. 12, 1803.

The first patient with influenza I was desired to visit, was on the 12th of February, but I have been informed it had made its appearance in this neighbourhood at rather an earlier period; during the months of March, April, and the beginning of May, it was most active, and in many instances that occurred under my observation, shewed symptoms of high pulmonary affection, where bleedings and vesicatories became expedient and salutary. It seemed principally to affect adults, and proved fatal to those only far advanced in life. It may not be improper to remark, that during the prevalence of this malady, I attended a maniacal patient; the family in which he lived consisted of eleven persons, ten had the influenza, and the majority of them in a severe manner; three constantly slept in the same room with this person during the whole progress of the disease, and experienced the full effects of its power, yet the catarrhal affection did not extend its influence in the least apparent degree to this patient. Whether this epidemic mic disease originated from atmospheric miasma, or from contagion, I will not undertake to elucidate; certain it is, I have known seven or eight persons fall suddenly ill the same day, in the same family; in others they have progressively sickened at the expiration of six, seven, or eight days; and in several instances I could enumerate, it shewed only a partial appearance, where several had it severely, while others escaped; none more observable than in my own family, three had it only out of six very slightly; and though I had been in the habit of visiting many patients daily, yet I was fortunately unworthy its attack.

